# Children and Adults With Mild COVID-19: Dynamics of the Memory T Cell Response up to 10 Months

**DOI:** 10.3389/fimmu.2022.817876

**Published:** 2022-02-07

**Authors:** Patricia Kaaijk, Verónica Olivo Pimentel, Maarten E. Emmelot, Martien C. M. Poelen, Alper Cevirgel, Rutger M. Schepp, Gerco den Hartog, Daphne F.M. Reukers, Lisa Beckers, Josine van Beek, Cécile A. C. M. van Els, Adam Meijer, Nynke Y. Rots, Jelle de Wit

**Affiliations:** ^1^ Centre for Infectious Disease Control, National Institute for Public Health and the Environment (RIVM), Bilthoven, Netherlands; ^2^ Faculty of Veterinary Medicine, Utrecht University, Utrecht, Netherlands

**Keywords:** SARS-CoV-2, COVID-19, mild symptoms, children, T cell immunity, adaptive immunity, antibody response, cytokines

## Abstract

**Background:**

Severe acute respiratory syndrome coronavirus-2 (SARS-CoV-2) has led to considerable morbidity/mortality worldwide, but most infections, especially among children, have a mild course. However, it remains largely unknown whether infected children develop cellular immune memory.

**Methods:**

To determine whether a memory T cell response is being developed, we performed a longitudinal assessment of the SARS-CoV-2-specific T cell response by IFN-γ ELISPOT and activation marker analyses of peripheral blood samples from unvaccinated children and adults with mild-to-moderate COVID-19.

**Results:**

Upon stimulation of PBMCs with heat-inactivated SARS-CoV-2 or overlapping peptides of spike (S-SARS-CoV-2) and nucleocapsid proteins, we found S-SARS-CoV-2-specific IFN-γ T cell responses in infected children (83%) and adults (100%) that were absent in unexposed controls. Frequencies of SARS-CoV-2-specific T cells were higher in infected adults, especially several cases with moderate symptoms, compared to infected children. The S-SARS-CoV-2 IFN-γ T cell response correlated with S1-SARS-CoV-2-specific serum antibody concentrations. Predominantly, effector memory CD4^+^ T cells of a Th1 phenotype were activated upon exposure to SARS-CoV-2 antigens. Frequencies of SARS-CoV-2-specific T cells were significantly reduced at 10 months after symptom onset, while S1-SARS-CoV-2-specific IgG concentrations were still detectable in 90% of all children and adults.

**Conclusions:**

Our data indicate that an antigen-specific T cell and antibody response is developed after mild SARS-CoV-2 infection in children and adults. It remains to be elucidated to what extent this SARS-CoV-2-specific response can contribute to an effective recall response after reinfection.

## Introduction

Tremendous research efforts have advanced our understanding of immunity to SARS-CoV-2. Most data on the immune response to SARS-CoV-2 was obtained from severe COVID-19 cases (1–4). However, the vast majority of infected individuals experience mild symptoms that do not require hospitalization (5–8). The question remains whether individuals, including children, with an asymptomatic or mild SARS-CoV-2 infection, develop immune memory, which may protect against subsequent SARS-CoV-2 infections. Persons with mild or asymptomatic infections often develop an antibody response, although not all cases do (8). It has been shown that SARS-CoV-2-induced antibody levels are waning over time (6, 9–11). On the other hand, T cell immunity is predicted to persist longer; after SARS-CoV infection in 2003, it was shown that T cell responses can persist for up to 17 years (12). Some studies investigated the T cell immunity induced after SARS-CoV-2 infection in mild symptomatic adult cases (6, 8, 13–16), showing weaker T cell responses in mild than in moderate or severe COVID-19 cases. CD4^+^ T cell responses against SARS-CoV-2 were more prominent than the CD8^+^ T cell response in adults with mild-to-moderate infection (8, 15, 16), while qualitatively impaired CD4^+^ T cell responses have been reported for critically ill patients (15).

Nevertheless, it remains unclear whether SARS-CoV-2 infection in children, usually showing a mild course, induces substantial T cell immunity. Only a few reports describe the immune responses in children with mild disease or asymptomatic infection, although in these studies T cells specifically reactive to SARS-CoV-2 were not investigated (17–20). Recently, a study was published investigating SARS-CoV-2 specific T cell responses in children (21). Induction of a sustainable T cell response is needed to provide immune memory for long-term protection against reinfections by facilitating an efficient and quick response upon re-exposure. Therefore, knowledge on the induction of memory T cell immunity after a mild course of SARS-CoV-2 infection in children and adults is useful for the consideration of the community mitigation measures needed to protect against COVID-19 and limit the spread of the virus.

In the present study, we examined the frequency and the phenotypic/functional characteristics of SARS-CoV-2-reactive T cells in infected children and adults with mild to moderate symptoms up to 10 months after symptom onset. In addition, T cell responses correlated with SARS-CoV-2-specific serum IgM, IgG, and IgA antibody concentrations.

## Material and Methods

### Clinical Studies

In this prospective cohort study, described previously (22), households were enrolled in which one adult (index case) tested PCR positive for SARS-CoV-2 between March-May, 2020. Blood samples were collected longitudinally from members of these households; from children (n=24) and adults (n=27) with PCR-confirmed SARS-CoV-2 infection (for details see **Table 1**). Disease severity was classified as follows: asymptomatic (absence of symptoms), mild (presence of at least one symptom such as cough, fever, loss of smell or taste, etc.; but an absence of symptoms indicative of lower airways infection), moderate (presence of shortness of breath with or without any other symptom, including hospitalized cases). In this cohort, none of the hospitalized patients were admitted to an intensive care unit, and therefore not considered severe cases. Three children remained asymptomatic during the study but tested PCR positive. Additionally, blood samples from age-matched unexposed children (n=13) and adults (n=12) were collected from two other cohort studies before the COVID-19 pandemic (respectively, 2018-2019 and 2009-2011). None of the study subjects were vaccinated against SARS-CoV-2.

**Table 1:** Demographic and clinical characteristics of adults (with mild versus moderate symptoms) and children with PCR confirmed SARS-CoV-2 infection and demographic data of unexposed participants.

Characteristics	Infected adults total n = 27	Infected adults mild n = 13	Infected adults moderate n = 14	Unexposed adults n = 12	Infected children n = 24	Unexposed children n = 13
Median age (range) – years	44 (18 – 87)	45 (23 – 87)	44 (18 – 54)	38 (20 – 51)	12 (2 – 16)	12 (2 – 16)
Sex – number (%) Male Female	14 (51.9) 13 (48.1)	8 (61.5) 5 (38.5)	6 (42.9) 8 (57.1)	6 (50.0) 6 (50.0)	11 (45.8) 13 (54.2)	5 (38.5) 8 (61.5)
Sign and symptoms – number (%) Any sign or symptom Fever Cough Chills Sore throat Runny nose Phlegm Headache Myalgia Fatigue Shortness of breath Loss of taste or smell Diarrhea	27 (100.0) 14 (51.9) 20 (74.1) 15 (55.6) 14 (51.9) 18 (66.7) 12 (44.4) 20 (74.1) 20 (74.1) 24 (88.9) 12 (44.4) 11 (40.7) 11 (40.7)	13 (100.0) 6 (46.2) 7 (53.8) 6 (46.2) 7 (53.8) 9 (69.2) 5 (38.5) 10 (76.9) 9 (69.2) 10 (76.9) 0 (0.0) 3 (23.1) 6 (46.2)	14 (100.0) 8 (57.1) 13 (92.9) 9 (64.3) 7 (50.0) 9 (64.3) 7 (50.0) 10 (71.4) 11 (78.6) 14 (100.0) 12 (85.7)* 8 (57.1) 5 (35.7)	N/A	21 (87.5) 6 (25.0) 11 (45.8) 2 (8.3) 3 (12.5) 15 (62.5) 2 (8.3) 7 (29.2) 1 (4.2) 6 (25.0) 2 (8.3) 3 (12.5) 2 (8.3)	N/A

*All moderate cases reported shortness of breath with the exception of two hospitalized cases.

n, number of subjects in specific group; N/A, not applicable.

The protocol for the SARS-CoV-2-related study, based on the WHO First Few Hundred (FFX) protocol, was approved by the Medical-Ethical Review Committee (MERC) of University Medical Center Utrecht (Netherlands Trial Register (https://www.trialregister.nl/): NL9850, MERC Reference number: NL13529.041.06). Protocols for the cohort studies with unexposed children (Immfact, NTR: NL9775, MERC Reference number: NL4679.094.13) and adults (NVI-255, NTR: NL1952, MERC Reference number: NL29241.000.09) (23) were approved by Medical-Ethical Review Committees of the Netherlands. Written informed consent was received from all participants and/or from parents/guardians of minor participants (<16 years old). All trial-related activities were conducted according to Good Clinical Practice, including the provisions of the Declaration of Helsinki.

### Peripheral Blood Mononuclear Cells and Serum Isolation

Peripheral blood mononuclear cells (PBMCs) were isolated from heparinized blood by centrifugation on a Ficoll-Hypaque gradient (Pharmacia Biotech) and cryopreserved at -135°C until use. Serum was separated from the blood by clotting and centrifugation and stored at -80°C until analysis.

### *Ex Vivo* Immune Profiling

Flow cytometry using Trucount tubes (BD Biosciences) was performed on fresh blood. Cells were stained for anti-human CD27 (O323), CD45 (HI30) and CD45RO (UCHL1) (all Biolegend) and CD3 (SK7), CD4 (SK3), CD8 (RPA-T8), CD38 (HIT2), and HLA-DR (G46-6) (all BD Bioscience), to analyze CD4^+^ and CD8^+^ T cells expressing CD38 and/or HLA-DR as markers for activated T cells.

Defrosted PBMCs from SARS-CoV-2 infected children (n=24) and adults (n=27), as well as from unexposed healthy children (n=13) and adults (n=12), were used for deep immune profiling by multicolor flow cytometry. (BD FACSymphony™). Cells were stained for anti-human CD3 (OKT3), CD14 (HCD14), CD28 (CD28.2), CD56 (5.1H11), CD57 (HNK-1), CD95 (DX2) and CCR7 (G043H7) (all Biolegend) and CD4 (RPA-T4), CD8 (RPA-T8), CD19 (SJ25C1), CD27 (L128), CD45RO (UCHL1) (all BD Bioscience) and eFluor 780 fixable viability stain (65-0865-14, ThermoFisher). For this purpose, major lymphocyte populations were discriminated by analyzing the CD3 expression to identify T cells, and identifying CD4^+^, CD8^+^, CD4^+^/CD8^+^ and CD4^-^/CD8^-^ T cells within the CD3^+^ T cells, analyzing CD19 expression to detect B cells, CD56 expression for NK cells and CD14 expression to identify cells of the myelomonocyte lineage. Memory CD4^+^ and CD8^+^ T cell subsets were further discriminated based on CD45RO and CCR7 staining (true naïve T cells (T_N_), CD45RO^-^, CD27^+^, CCR7^+^, CD95^-^; central memory T cells (T_CM_), CD45RO^+^, CD27^+^; effector memory T cells (T_EM_), CD45RO^+^, CD27^-^; terminally differentiated effector memory T cells re‐expressing CD45RA (T_EMRA_), CD45RO^-^, CD27^-^, CD28^-^, CD57^+^).

Flow cytometry data analysis was performed using FlowJo software, version 10 (TreeStar).

### Generation of Heat-Inactivated Virus Stocks of SARS-CoV-2

SARS-CoV-2 isolate, hCoV-19/Netherlands/Zuid_Holland_0133R/2020, was obtained from a Dutch patient. Virus was grown on VERO-E6 cells in DMEM medium (Gibco; Thermo Fisher Scientific) supplemented with 1x penicillin-streptomycin-glutamine (Gibco) and 2% FBS for approximately 48 hours under BSL-3 conditions. At >90% cytopathic effect (CPE), the suspension was collected and spun down (4000 × g, 10 min) to remove cell debris. Virus stocks were aliquoted and stored at −80°C until use. The 50% tissue culture infective dose (TCID_50_), 7.63.10^7^ TCID_50_/ml, was determined by the Reed and Muench method. Heat-inactivation was performed by incubating the virus at 60°C for 2 hours, after which the inactivated virus stocks were stored at −80°C until use.

### Interferon Gamma ELISPOT

Multiscreen filtration ELISPOT plates (Millipore, Merck) were prewetted with 35% ethanol for ≤1 minute and washed with sterile water and PBS. Plates were coated with 5 μg/mL anti-human IFN-γ antibodies (1-D1K, Mabtech) overnight (4°C), then washed with PBS. PBMCs were incubated with heat-inactivated SARS-CoV-2 (MOI-3), or 15-mers overlapping peptides (11 amino acids overlap) covering whole spike protein of SARS-CoV-2 (S-SARS-CoV-2), whole nucleocapsid protein (N-SARS-CoV-2), or S-HCoV-OC43 (0.1 µM/peptide, all JPT), seeded on ELISPOT plates (2.10^5^ cells/well), and incubated for 20 hours, 37°C, 5% CO2 in 100 µl AIM-V (Lonza) with 2% human serum (Sigma). DMSO and PHA (Sigma) were negative and positive controls, respectively. Subsequently, plates were washed and incubated for 1 hour with 1 μg/mL anti-human IFN-γ detection biotinylated-antibody (7-B6-1, Mabtech) in PBS-0.05% casein (Sigma). Plates were washed and incubated with Streptavidin-poly-HRP (Sanquin) in PBS-0.05% casein for 1 hour. After washing, plates were developed with TMB substrate (Mabtech). Spots were analyzed with CTL software. The number of spots from negative controls was subtracted from total spot numbers induced by antigen-specific stimulation; more than 5 spots, after background subtraction, were considered positive.

### Immunophenotyping and Expression of Activation Markers After *In Vitro* Stimulation

Activated T cells were determined by harvesting cells from the IFN-γ ELISPOT plates and subsequently flow cytometric analysis was performed using activation markers. Cells were stained for anti-human CD3 (SK7), CD4 (SK3), CD8 (RPA-T8), CD45RO (UCHL1), CD25 (2A3), CD56 (NCAM16.2), CD69 (FN50), OX40 (L106)and fixable viability stain 780 (BD Bioscience) and CCR7 (G043H7) (Biolegend). After fixation and permeabilization, using FoxP3/Transcription Factor Staining Buffer Set (eBioscience, Thermo Fisher Scientific, Waltham, Mass) cells were stained intracellularly for anti-human, CD137 (4-1BB) and CD154 (BD Bioscience). Data were acquired on a FACS Symphony analyzer (BD) and analyzed using FlowJo (V10, Tree Star, Ashland, Ore).

### Antibody Assays

IgM, IgG, and IgA concentrations against SARS-CoV-2 monomeric spike-S1 (40591-V08H; Sino Biological) were determined in serum using a fluorescent bead-based multiplex immunoassay (MIA) as published previously (24), with previously determined cut-off values for seroprevalence of 1.20, 1.04, and 0.50 AU/mL for respectively SARS-CoV-2 monomeric spike-S1-specific IgM, IgG and IgA concentrations (10).

### Cytokine Release Assay

Cell-free culture supernatants were harvested from the IFN-γ ELISPOT plates and analyzed using a bead-based multiplex immunoassay (MIA) quantitating levels of IL-2, IL-4, IL-5, IL-6, IL-9, IL-10, TNF, IL-13, IL-17A, IL-17F, IL-22 (LEGENDplex; BioLegend) according to the manufacturer’s instructions and using FACSCanto (BD). For analysis, the online cloud-based program, The LEGENDplex™ Data Analysis Software Suite, was used. The minimum detection threshold (MDT) for each cytokine as calculated by the manufacturer was: 1.4 pg/ml for IL-2, 0.9 pg/ml for IL-4, 1.3 pg/ml for IL-5, 1.1 pg/ml for IL-6, 1.5 pg/ml for IL-9, 0.9 pg/ml for IL-10, 0.9 pg/ml for TNF, 1.4 pg/ml for IL-13, 2.0 pg/ml for IL-17A, 1.0 pg/ml for IL-17F, and 1.5 pg/ml for IL-22. Background signal for IL-6 and TNF from unstimulated controls was as high as stimulated samples, therefore these cytokines were excluded from analysis.

### Statistics

Statistical analyses were performed using GraphPad Prism version 9.1.0. For unpaired comparisons, Mann-Whitney U test (two groups) or Kruskal-Wallis rank-sum test with Dunn’s *post hoc* test (≥3 groups) were used. Paired data were compared using the Wilcoxon signed-rank test (two groups) or the Friedman test with Dunn’s multiple comparison test (≥3 groups). Median values for paired comparisons were calculated from subjects with complete data for all time points. Correlation coefficients (r_s_) were determined with Spearman’s rank correlation. Non-parametric tests were used since data were mostly non-normally distributed according to the Shapiro-Wilk test. P values <0.05 were considered significant.

## Results

### Study Subjects

SARS-CoV-2 specific T cell responses were assessed from SARS-CoV-2-infected children and adults, and for comparison from unexposed children and adults. None of the study subjects were vaccinated against SARS-CoV-2. Demographic and clinical characteristics are presented in **Table 1**. Blood samples from 24 children and 27 adults (13 with mild symptoms and 14 with moderate symptoms, including two hospitalized cases) with PCR-confirmed SARS-CoV-2 infection were collected. The median time point of the first sample (T1) was for children 8 days [5.0-16 days] and for adults 12.5 days [interquartile range (IQR) 11-14 days] post-symptom onset. Additional blood samples were taken 10-14 days after T1 (referred to as ‘T2’) and only for adults also at 4-6 weeks after T1 (referred to as ‘T3’).

### *Ex Vivo* Determination of Activated T Cells Over Time After SARS-CoV-2 Infection in Children and Adults

No significant differences in major immune cell types were found over time after infection neither in children nor adults, i.e. frequencies of total T cells, B cells, monocytes, or NK cells were comparable between infected groups and healthy age-matched unexposed groups (**Supplementary Figure 1**). In infected adults, slightly higher frequencies of CD4^+^ T_EM_ (T_EM_; CD45RO^+^, CD27^-^) cell subsets were found at T3 compared to T1 (4.7% vs 3.5%; P= 0.0061), but there was no significant difference in the frequency of the T_EM_ subset of these time points and the uninfected controls (3.6%). Frequencies of CD8^+^ T_EM_ cells of infected adults were slightly higher at T2 compared to T1 (4.2% vs 3.8%; P= 0.041). No differences in memory T cell subsets were found between mild and moderate COVID-19 adults cases nor in children over time after infection.

The *ex vivo* expression of CD38 and HLA-DR on T cells was determined as a measure for antigen-specific activation after SARS-CoV-2 infection. In children, no changes in CD38/HLA-DR co-expression on CD4^+^ T cells were found between different time points after infection (**Supplementary Figure 2A**, upper panel). In SARS-CoV-2-infected adults, a transiently higher percentage of cells expressing CD38/HLA-DR was observed within the CD4^+^ T central memory (T_CM_; CD45RO^+^, CD27^+^) subset (1.1% at T1) that declined over time after infection (to 0.55% at T2, and to 0.37% at T3) and within the CD4^+^ terminally differentiated effector memory re‐expressing CD45RA (T_EMRA_; CD45RO^-^, CD27^-^, CD28^-^, CD57^+^) cell subset (0.58% at T1) that declined to 0.0% at both T2 and T3 (**Supplementary Figure 2B**, upper panel). In both children and adults, a higher frequency of CD8^+^/CD38^+^/HLA-DR^+^ T cells within the T_CM_/T_EM_/T naïve (T_N_; CD45RO^-^, CD27^+^, CCR7^+^, CD95^-^) subsets were observed at T1 [4.1%/4.9%/0.33% (children) and 5.5%/3.1%/1.0% (adults)] that declined in time after infection (to 2.7%/2.1%/0.17% (children) and 2.7%/1.7%/0.30% (adults) at T2 and to 2.2%/0.81%/0.23% (adults) at T3 (**Supplementary Figures 2A, B**, lower panels).

### SARS-CoV-2 Specific IFN-γ^+^ T Cell Response

**Figure 1:**
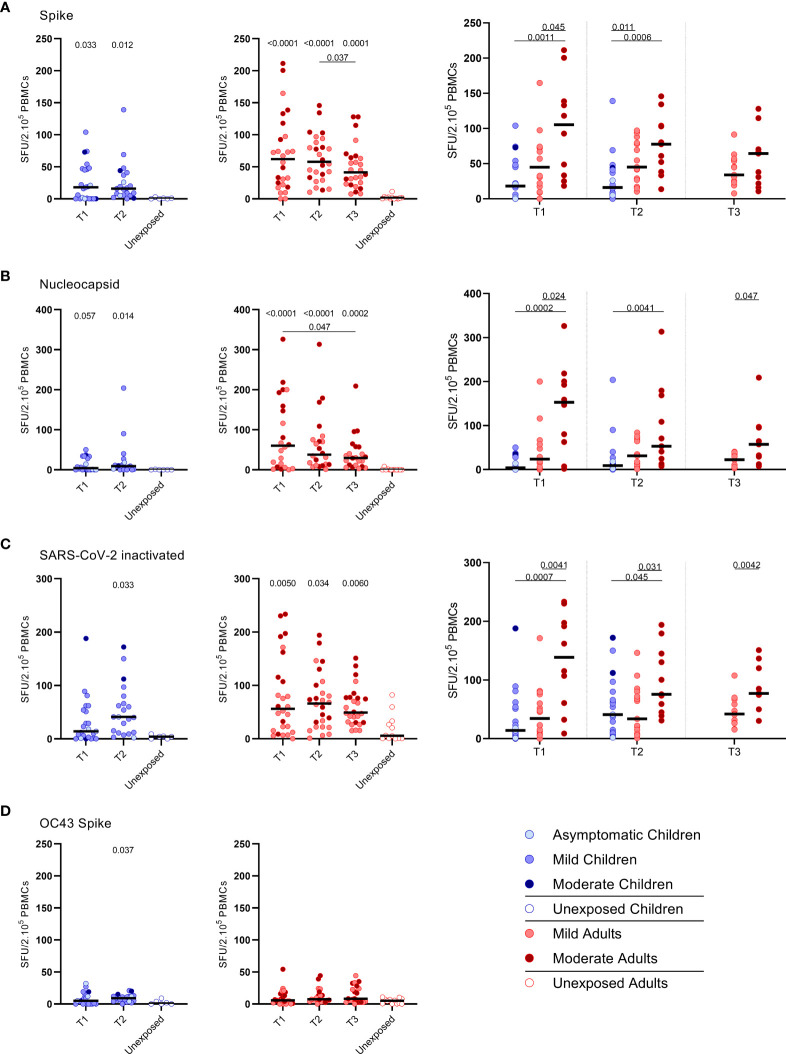
SARS-CoV-2-specific IFN-γ^+^ T cell response in infected children and infected adults (mild and moderate cases) versus unexposed healthy controls over time after infection. Dot plots summarizing the frequencies of IFN-γ-producing cells responding to SARS-CoV-2 and HCoV-OC43 antigens for **(A–D)** children (left panel, blue), adults (middle panel, red), and children versus mild and moderate adult cases separately (right panel), over time after infection, and compared to unexposed adults/children (ELISPOT assay). Frequencies of IFN-γ-producing cells responding to **(A)** set of overlapping peptides of SARS-CoV-2 spike protein, **(B)** set of overlapping peptides of SARS-CoV-2 nucleocapsid protein, **(C)** inactivated SARS-CoV-2, and **(D)** set of overlapping peptides of HCoV-OC43 spike protein. Each dot represents one subject. Bars indicate the median of spot-forming units per 200,000 PBMCs. SFU, spot-forming unit. **(A–D)** P values related to comparisons with the unexposed controls are listed at the top of the graph, above the corresponding group for comparison. For unpaired comparisons, Mann-Whitney U test (two-group comparisons) (mild adults versus moderate adults) or Kruskal-Wallis rank-sum test with Dunn’s *post hoc* test for multiple comparisons were used (children versus mild adults versus moderate adults; unexposed versus infected children or adults at T1 versus T2 versus T3). Differences between paired data were compared using the Wilcoxon signed-rank test (for comparison of two paired groups) (infected children at T1 versus T2) or the Friedman test with Dunn’s multiple comparison tests (infected adults at T1 versus T2 versus T3). Statistically significant comparisons are indicated, with P values < 0.05 considered significant. T1, first timepoint of sampling for adults median 12.5 days and children median 8 days post-symptom onset; T2, 10-14 days after T1; T3, 4-6 weeks after T1.

IFN‐γ^+^‐producing cells were detected by ELISPOT upon stimulation with overlapping peptides covering spike protein (S) of SARS-CoV-2 (S-SARS-CoV-2) in 83% (20/24) of infected children and in 100% (27/27) of infected adults, whereas IFN γ^+^ responses were found in 0% (0/6) and 8.3% (1/12) of the unexposed children and adults, respectively. Frequencies of SARS-CoV-2-specific IFN-γ^+^ T cells were lower in infected children than in infected adults (**Figures 1A–C**); the median spot forming units (SFU)/2.10^5^ PBMCs for infected children versus infected adults was 18 versus 62 (P=0.0021) at T1 upon stimulation with S-SARS-CoV-2. On average, a 2-fold higher frequency of SARS-CoV-2-specific IFN-γ^+^ T cells was found in adults with moderate symptoms compared to mild symptomatic adults for the three time points. For all antigenic stimuli used (i.e. S-SARS-CoV-2, overlapping peptides covering nucleocapsid protein (N) of SARS-CoV-2 (N-SARS-CoV-2), or inactivatedwhole SARS-CoV-2), the moderately ill adults had significantly higher frequencies of IFN-γ-producing T cells compared tochildren at T1. The mild symptomatic adults also generally showed higher frequencies of SARS-CoV-2-specific IFN-γ^+^ Tcells than infected children, although not statistically significant for inactivated whole SARS-CoV-2 (**Figures 1A–C**).

The pre-existing T cell response against S-HCoV-OC43 of infected children was very low, although S-HCoV-OC43-specific T cell frequency was slightly higher at T2 compared to the agematched unexposed control group (9.0 versus 1.0 SFU/2.105 PBMCs; P=0.037) (**Figure 1D**, left panel). In infected adults, no difference in numbers of S-HCoV-OC43-specific T cells was found between SARS-CoV-2-infected and unexposed adults (**Figure 1D**, right panel). In both SARS-CoV-2-infected children and adults, the frequency of IFN-γ^+^ T cells was 4 to 60-fold lower after stimulation with S-HCoV-OC43 (**Figure 1D**) compared to stimulation with any of the SARS-CoV-2 antigens (**Figures 1A–C**). No significant difference in frequency of SHCoV-OC43-reactive T cells was observed between unexposed adults and unexposed children or between mild and moderate COVID-19 cases.

### Activation of Effector Memory CD4^+^ T Cells Upon SARS-CoV-2-Specific Stimulation

PBMCs were harvested from the IFN-γ ELISPOT plates to determine whether mainly CD4+ or CD8+ T cell populations became activated upon SARS-CoV-2-specific stimulation. For this purpose, the expression of the following activation markers were analyzed by flow cytometry: CD25, CD137, CD154, CD69, OX40 (25–30). Especially the CD25 (IL-2Rα) and CD137 (4-1BB) co-expression on T cells of infected subjects increased significantly upon the various SARS-CoV-2 antigenic stimulations compared to mock stimulation (**Supplementary Figure 3**). Although analysis of CD137/CD25 co-expression is generally not used by default, in the present study we used this to identify SARS-CoV-2 antigen-specific T cells.

**Figure 2:**
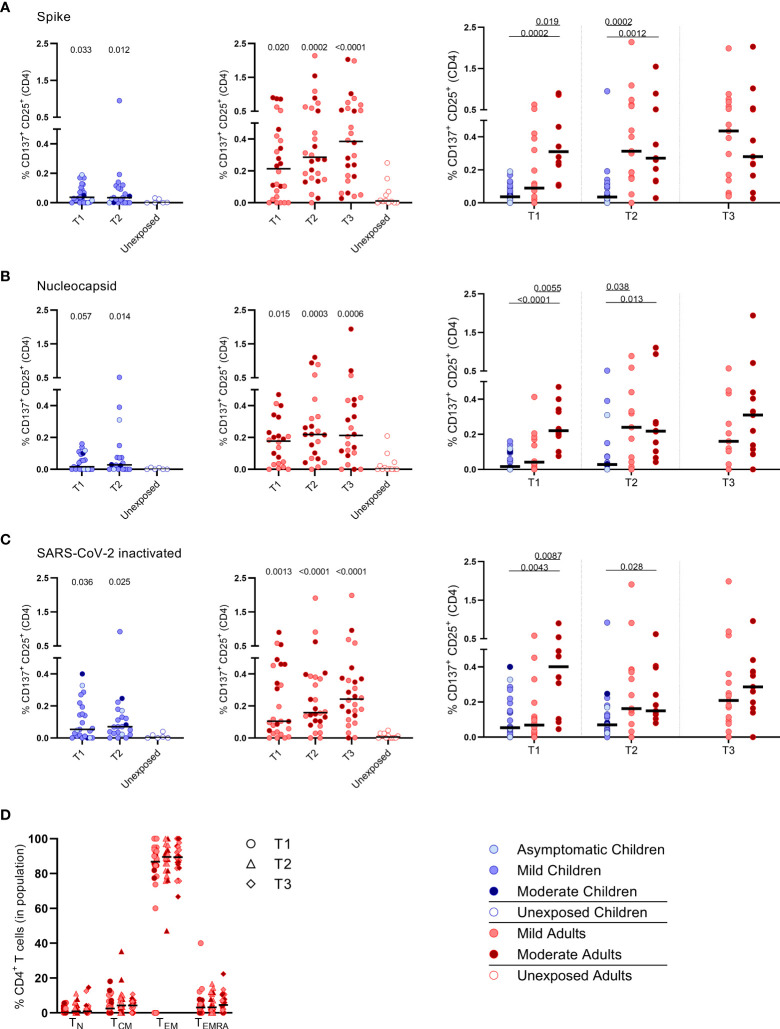
Frequencies of activated CD4^+^ T cells of infected children and infected adults (mild and moderate cases) versus unexposed healthy controls over time after infection. Dot plots summarizing the percentages of CD25^+^/CD137^+^ activated CD4^+^ T cells responding to SARS-CoV-2 antigens for **(A–D)** children (left panel, blue), adults (middle panel, red), and children versus mild and moderate adult cases separately (right panel). Percentages of CD25^+^/CD137^+^ activated CD4^+^ T cells responding to **(A)** set of overlapping peptides of SARS-CoV-2 spike protein, **(B)** set of overlapping peptides of SARS-CoV-2 nucleocapsid, **(C)** inactivated SARS-CoV-2. **(D)** Immunophenotyping at the single-cell level showing the different memory subsets within the SARS-CoV-2-specific activated CD4^+^ T cells from infected adults. Each dot represents one subject. Bars indicate the median percentage of total CD4^+^ T cells. **(A–C)** P values related to comparisons with the unexposed controls are listed at the top of the graph, above the corresponding group for comparison. For unpaired comparisons, Mann-Whitney U test (two-group comparisons) (mild adults versus moderate adults) or Kruskal-Wallis rank-sum test with Dunn’s *post hoc* test for multiple comparisons were used (children versus mild adults versus moderate adults; unexposed versus infected children or adults at T1 versus T2 versus T3). Differences between paired data were compared using the Wilcoxon signed-rank test (for comparison of two paired groups) (infected children at T1 versus T2) or the Friedman test with Dunn’s multiple comparison tests (infected adults at T1 versus T2 versus T3). Statistically significant comparisons are indicated, with P values < 0.05 considered significant. T1, first timepoint of sampling for adults median 12.5 days and children median 8 days post-symptom onset; T2, 10-14 days after T1; T3, 4-6 weeks after T1.

Primarily CD4^+^ T cells and not CD8^+^ T cells expressed CD25/CD137 activation markers upon SARS-CoV-2 antigenic stimulation, in infected subjects (**Figure 2** and **Supplementary Figure 4**). Compared to the unexposed age-matched groups, higher frequencies of activated CD4^+^ T cells were observed in both infected children and infected adults after stimulation with any of the three SARS-CoV-2 antigen preparations at T1. Frequencies of SARS-CoV-2-specific activated CD4^+^ T cells were significantly lower in infected children than in adults (upon S-SARS-CoV-2-specific stimulation, 0.04% CD25^+^/CD137^+^ T cells of total CD4^+^ T cells for infected children versus 0.21% for infected adults at T1; P=0.0034). Frequencies of SARS-CoV-2-specific activated CD4^+^ T cells, irrespective of used SARS-CoV-2 antigen, were comparable between adults with moderate COVID-19 illness and mildly symptomatic adults. The observed SARS-CoV-2-specific activated CD4^+^ T cells were mainly effector memory (CD45RO^+^/CCR7^-^) (TEM) (**Figure 2D**).

### Correlations Between SARS-CoV-2-Specific IFN-γ^+^ T Cell Responses and CD4^+^ T Cell Response

**Figure 3:**
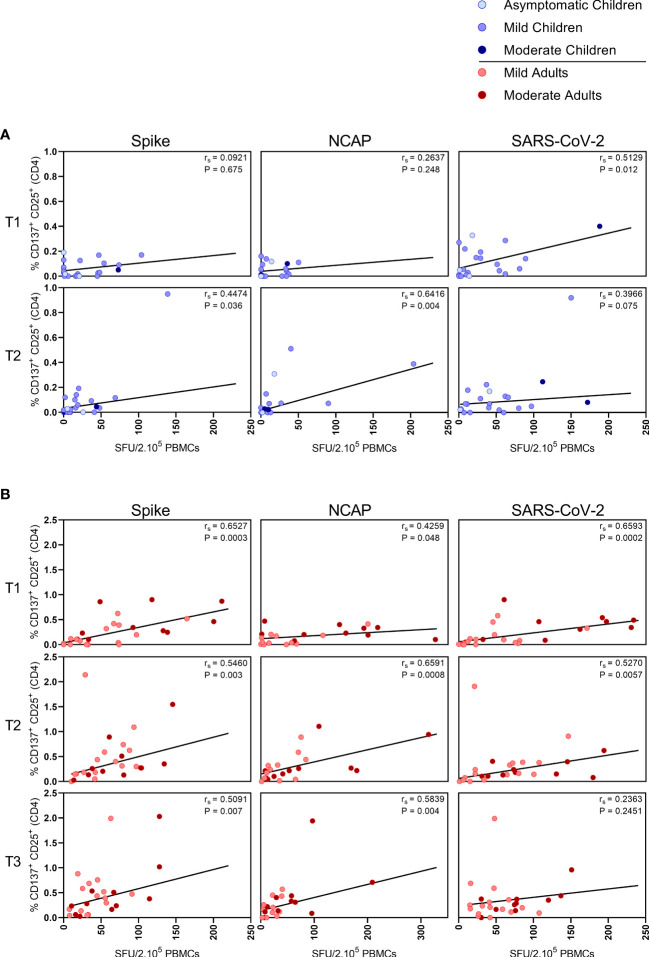
Correlation between IFN-γ^+^ T cell frequency and activated CD4^+^ T cells. Spearman correlation between frequency of IFN-γ^+^ responder cells and percentages of CD25^+^/CD137^+^ activated CD4^+^ T cells of **(A)** children and **(B)** adults responding to a set of overlapping peptides of SARS-CoV-2 spike protein (left panel), or a set of overlapping peptides of SARS-CoV-2 nucleocapsid protein (middle panel) or inactivated SARS-CoV-2 (right panel) at different time points after infection. Each dot represents one subject. Correlation coefficients (r_s_) were determined with Spearman’s rank correlation. P values < 0.05 were considered significant. T1, first timepoint of sampling for adults median 12.5 days and children median 8 days post-symptom onset; T2, 10-14 days after T1; T3, 4-6 weeks after T1.

In children,moderate correlations were observed between IFN-γ^+^ T cell frequency and activated (CD137^+^ CD25^+^) CD4^+^ T cells after stimulation with N-SARS-CoV-2 (r_s_=0.64; P=0.004) at T2, and at T1 after stimulation with inactivated SARS-CoV-2 (r_s_=0.51; P=0.012) (**Figure 3A**). In adults, IFN-γ^+^ T cell frequency and activated (CD137^+^ CD25^+^) CD4^+^ T cells correlated after stimulation with both S-SARS-CoV-2 and N-SARS-CoV-2 at all three time points after infection (rs ranging between 0.51-0.72) and after stimulation with inactivated SARS-CoV-2 at T1 and T2 (respectively, r_s_=0.70 and r_s_=0.53) (**Figure 3B**).

### SARS-CoV-2-Specific Release of Cytokines

**Figure 4:**
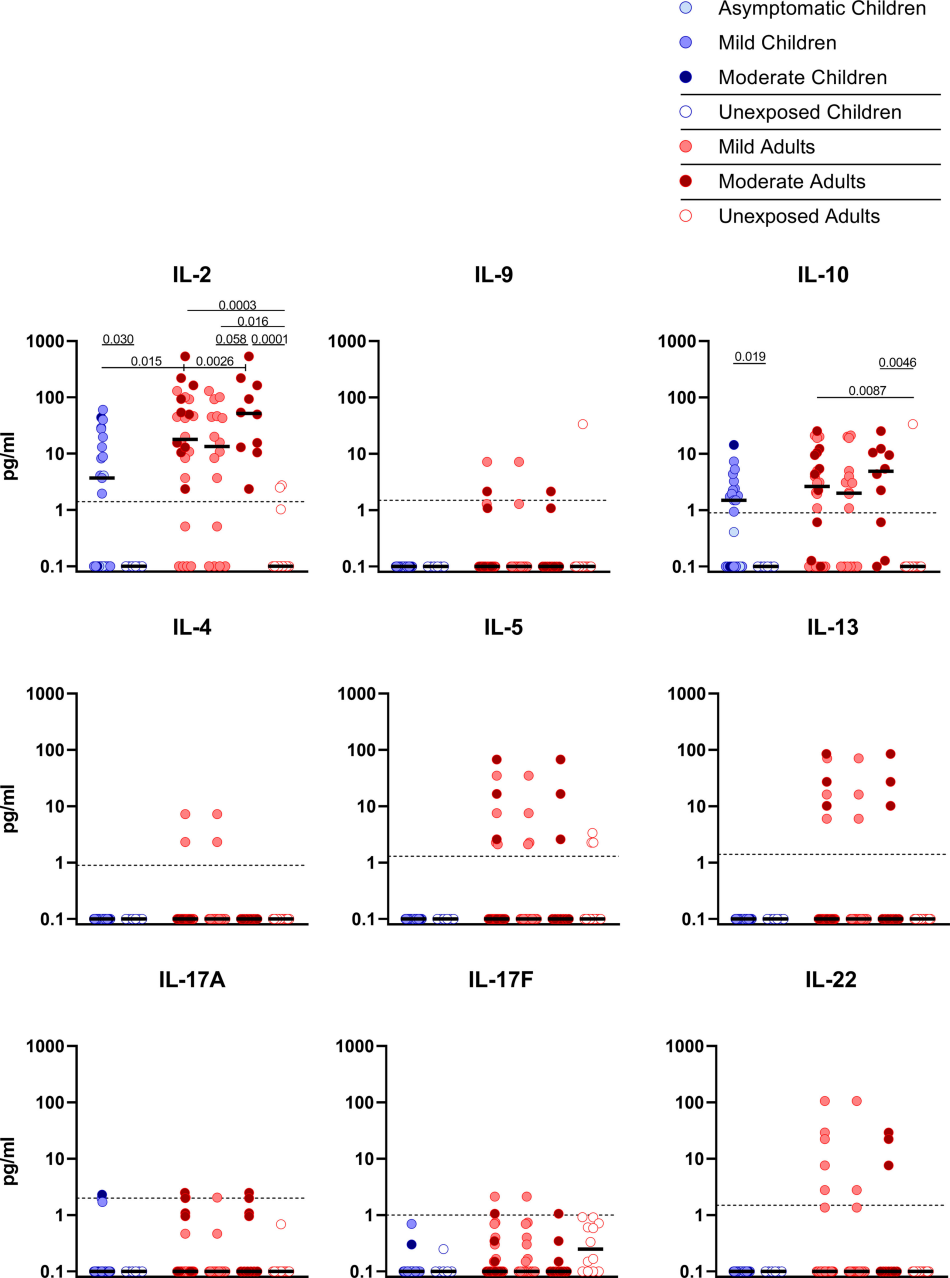
Cytokine release in infected children and mild versus moderate symptomatic adults over time after infection. Cell-free culture supernatants were harvested from IFN-γ ELISPOT plates and the release of the following cytokines was measured in T1 samples: IL-2, IL-4, IL-5, IL-9, IL-10, IL-13, IL-17A, IL-17F, IL-22. Dot plots show the concentration of cytokines (pg/ml) after stimulation of PBMCs with a set of overlapping peptides of SARS-CoV-2 spike protein. Infected children and unexposed children versus infected adults with mild or moderate disease and unexposed adults are depicted. Minimum detection threshold (MDT) concentrations for each cytokine, as calculated by the manufacturer and mentioned in the Material and Methods section, are indicated with horizontal dotted lines. Each dot represents one subject. Bars indicate the median cytokine concentration (pg/ml). For two-group comparisons (infected children versus unexposed children), Mann-Whitney U test was used. Kruskal-Wallis rank-sum test with Dunn’s *post hoc* test for multiple comparisons was used (all adults versus mild adults versus moderate adults versus unexposed adults; all adults versus mild adults versus moderate adults versus infected children). P-values ≤ 0.05 are presented.

Upon stimulation with S-SARS-CoV-2, PBMCs from SARS-CoV-2-infected children secreted more IL-2 than unexposed children, albeit at very low amounts (3.7 versus 0.1 pg/ml; P=0.030). Similar trends were observed in SARS-CoV-2-infected adults compared to unexposed adults, for both IL-2 (18.0 versus 0.1 pg/ml; P=0.0003). S-SARS-CoV-2-specific IL-2 secretion was higher in infected adults compared to infected children (P=0.015). However, IL-2secretion was only significantly higher in adults with moderate COVID-19 (51.8 versus 3.7 pg/ml; P=0.023) and not in adults with mild symptoms compared to infected children (**Figure 4**). Other cytokines were only secreted at very low levels and not significantly.

### Correlations Between SARS-CoV-2-Specific IFN-γ^+^ T Cell Frequency and Antibody Response

**Figure 5:**
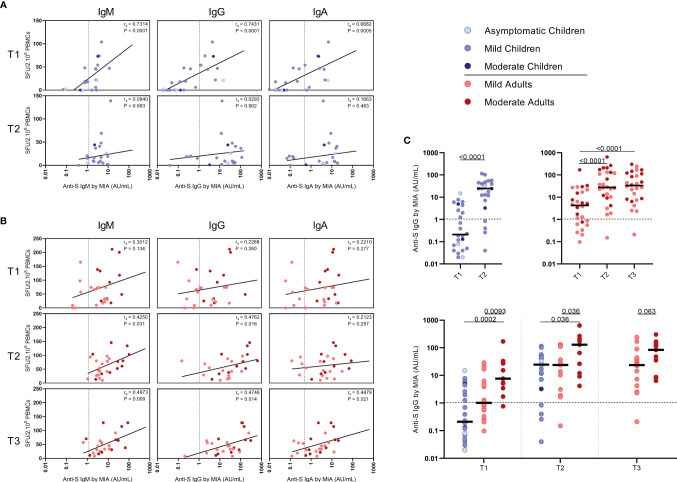
Correlation between frequencies of SARS-CoV-2-specific IFN-γ^+^ T cells and antibody concentrations to S1-SARS-CoV-2. Spearman correlation between frequency of SARS-CoV-2-specific IFN-γ^+^ responder cells and concentrations of spike-S1 IgM, IgG, or IgA serum antibodies measured by multiplex immunoassay (MIA) at different time points after infection in **(A)** children and **(B)** adults. Concentrations of spike-S1 IgG serum antibodies at the various time points after SARS-CoV-2 infection in the whole group of children and adults (**C**, upper panel) and in the whole group of children versus the mild and moderate adult cases (**C**, lower panel). Cut-off values for seroprevalence are indicated with vertical or horizontal dotted lines. Each dot represents one subject. Correlation coefficients (r_s_) were determined with Spearman’s rank correlation. P values < 0.05 were considered significant. Differences in IgG concentrations for paired data were compared using the Wilcoxon signed-rank test (for comparison of two paired groups) (infected children at T1 versus T2) or the Friedman test with Dunn’s multiple comparison tests (infected adults at T1 versus T2 versus T3). For unpaired comparisons, Mann-Whitney U test (two-group comparisons) (mild adults versus moderate adults) or Kruskal-Wallis rank-sum test with Dunn’s *post hoc* test for multiple comparisons were used (children versus mild adults versus moderate adults; at T1 versus T2 versus T3). Statistically significant comparisons are indicated, with P values < 0.05 considered significant. T1, first timepoint of sampling for adults median 12.5 days and children median 8 days post-symptom onset; T2, 10-14 days after T1; T3, 4-6 weeks after T1.

Serum antibody concentrations against the SARS-CoV-2 spike S1 protein (S1-SARS-CoV-2) above the previously established cutoff level (10) at any of the two sampling time points were found in 83.3% (IgM), 79.2% (IgG), and 75.0% (IgA) of the infected children. From the four children without detectable S-SARS-CoV-2-specific T cell responses, two did have S1-SARS-CoV-2-specific IgM, IgG, and IgA antibodies; the other two did not. In adults, 100%, 96.3%, and 88.9% were seropositive for respectively, IgM, IgG, and IgA antibodies to S1-SARS-CoV-2 at any of the three sampling time points. Interestingly, in children good correlations were observed between S-SARS-CoV-2 IFN-γ^+^ T cell frequency and S1-SARS-CoV-2-specific serum IgM, IgG and IgA concentrations, though this was only observed at T1 [for IgM, R_s_=0.73 (P<0.0001); IgG, r_s_=0.74 (P<0.0001); IgA, r_s_=0.68 (P=0.0004)] (**Figure 5A**). In adults, frequency of S-SARS-CoV-2-specific T cells was also correlated with S1-SARS-CoV-2-specific serum IgM concentrations at T2 and T3 [respectively, r_s_=0.42 (P=0.03) and r_s_=0.50 (P=0.01)], with S1-SARS-CoV-2-specific serum IgG concentrations at T3 [r_s_=0.47 (P=0.01)], and with S1-SARS-CoV-2-specific serum IgA concentrations at T3 (r_s_=0.45 (P=0.02)) (**Figure 5B**).

The median S1-SARS-CoV-2-specific serum IgG concentrations (**Figure 5C**) for infected children increased >100-fold in time after infection, from 0.21 AU/ml at T1 to 25 AU/ml at T2, while a 7-fold increase was observed for infected adults from T1 (median IgG concentration of 3.9 AU/ml) to T2 (26 AU/ml)), and an 8-fold increase from T1 to T3 (31 AU/ml) (**Figure 5C**, upper panel). Nevertheless, S1-SARS-CoV-2-specific serum IgG concentrations of infected adults experiencing moderate COVID-19 were clearly higher than levels of infected children or adults with mild symptoms at all time points after infection (**Figure 5C**, lower panel).

### SARS-CoV-2-Specific IFN-γ^+^ T Cell Frequency and Antibody Response at 10 Months After Infection

To study the long-term SARS-CoV-2-specific immune response, we collected follow-up blood samples at 10 months ± 13 days after symptoms onset (referred to as ‘T4’) from 18 children and 21 adults. Vaccinated persons (adults: n=3) at the time of T4 sampling were excluded from analysis. In children, the median age was 12 years (5-16 years) and 56% were female at T4 sampling. The median age of adults was 44 years (18-87 years), 48% were female, and 48% were mild cases at T4 sampling.

**Figure 6:**
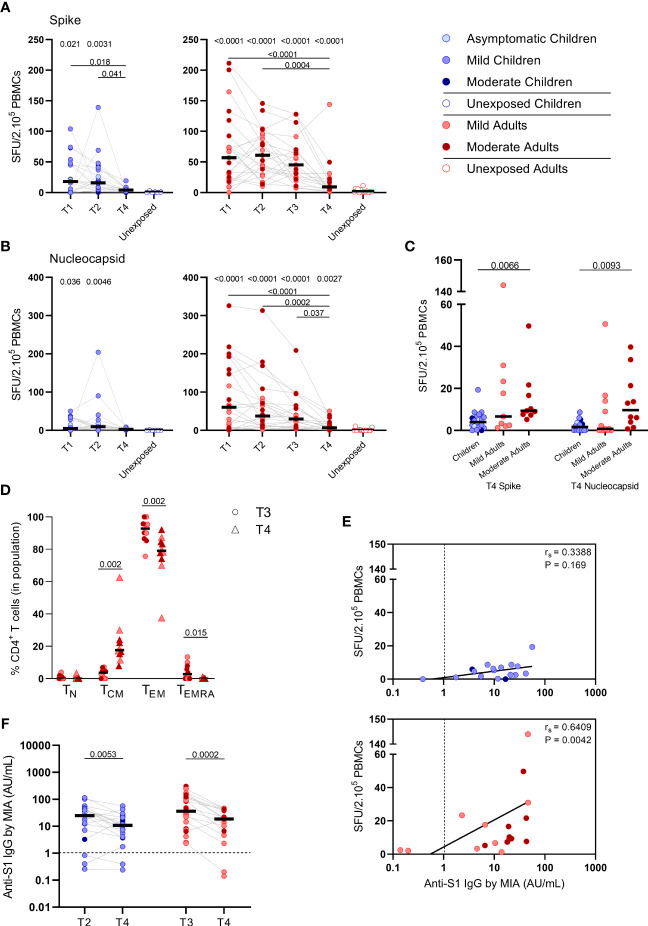
SARS-CoV-2-specific immune response up to 10 months after infection. Dot plots summarizing the frequencies of IFN-γ-producing cells by ELISPOT assay responding to **(A)** a set of overlapping peptides of SARS-CoV-2 spike protein and **(B)** a set of overlapping peptides of SARS-CoV-2 nucleocapsid protein in children (left panel, blue) and adults (right panel, red), at different time points after infection, and compared to unexposed children/adults. **(C)** Dot plots showing the frequencies of IFN-γ-producing cells responding to SARS-CoV-2 spike and nucleocapsid proteins, at 10 months after symptom onset (T4) comparison of groups of children versus total, mild, and moderate adult cases separately. **(D)** Immunophenotyping at the single-cell level comparing the frequencies of different memory subsets from T3 versus T4 within the SARS-CoV-2-specific activated CD4^+^ T cells from infected adults. **(E)** Spearman correlation between frequency of IFN-γ^+^ responder cells and concentrations of anti-spike-S1 IgG serum antibodies measured by multiplex immunoassay (MIA) at T4 in children (upper panel, blue) and adults (lower panel, red). **(F)** Dot plots comparing the concentrations of anti-spike-S1 IgG serum antibodies at T2 or T3 versus T4 in children and adults. Each dot represents one subject. Bars indicate the median. SFU, spot-forming unit. **(A, B)** P values related to comparisons with the unexposed controls are listed at the top of the graph, above the corresponding group for comparison. For unpaired comparisons, Mann-Whitney U test (two-group comparisons) (mild adults versus moderate adults, infected children or adults at T4 versus unexposed children or adults) or Kruskal-Wallis rank-sum test with Dunn’s *post hoc* test for multiple comparisons were used (children versus total adults versus mild adults versus moderate adults; unexposed versus infected children or adults at T1 versus T2 versus T3). Differences between paired data were compared using the Wilcoxon signed-rank test (for comparison of two paired groups) (memory T cell subsets at T3 versus T4; concentrations of anti-spike-S1 IgG serum antibodies at T2 or T3 versus T4) or the Friedman test with Dunn’s multiple comparison tests (infected children or adults at T1 versus T2 versus T3 versus T4). Statistically significant comparisons are indicated, with P values < 0.05 considered significant. Cut-off values for seroprevalence are indicated with vertical dotted lines in **(E, F)**. Correlation coefficients (r_s_) were determined with Spearman’s rank correlation. Statistically significant comparisons are indicated. P values < 0.05 were considered significant. T1, first timepoint of sampling for adults median 12.5 days and children median 8 days post-symptom onset; T2, 10-14 days after T1; T3, 4-6 weeks after T1; T4, 10 months ± 13 days after symptom onset.

S-SARS-CoV-2-specific IFN-γ^+^ responses higher than 5 SFU/2.10^5^ PBMCswere still found in 44% (8/18) of the infected children and 81% (17/21) of the infected adults at 10 months after symptom onset. In children, frequencies of S-SARS-CoV-2-specific IFN-g^+^ T cells underwent a 4-fold decline at 10 months (T4) compared to frequencies at 3 weeks (T2, P=0.027) after infection, and were no longer significantly different from unexposed children (**Figure 6A**, left panel). Also in adults, IFN-γ^+^ S-SARS-CoV-2-specific T cell frequencieswere 6 to 7-fold lower at 10 months compared to earlier time points after infection. In contrast to children, frequencies of SSARS-CoV-2-specific IFN-γ^+^ T cells at T4 were still significantly higher in infected adults compared to unexposed adults (9.2 versus 1.7 SFU/2.10^5^ PBMCs; P=0.0003) (**Figure 6A**, right panel). IFN-γ^+^ N-SARS-CoV-2-specific T cell frequencies also declined at 10 months upon infection in adults compared to earlier time points (7.7 SFU/2.10^5^ PBMCs at T4 vs 62 (P<0.0001), 47.0 (P=0.0008), and 31.1 (P=0.039) SFU/per 2.10^5^ PBMCs at T1, T2, and T3, respectively), but remained significantly higher compared to unexposed adults (0.05 SFU/2.10^5^ PBMCs) (**Figure 6B**, right panel). Frequencies of IFN-γ^+^ N-SARS-CoV-2-specific T cells were low in children and no changes over time were observed (**Figure 6B**, left panel). Similar to earlier time points, S-SARS-CoV-2-specific responses were 2.3-fold higher in adults compared to children at 10 months after infection (9.2 versus 4.0 SFU/2.10^5^ SFU/PBMCs). These differences were mainly caused by slightly higher responses from adults experiencing moderate symptoms (P=0.0021) (**Figure 6C**). Albeit not significant, a trend of higher frequencies of S-SARS-CoV-2-specific IFN-γ+ T cells in infected adults with moderate symptoms persisted at 10 months compared to mild disease (**Figure 6C**). In line with results from earlier time points, the SARS-CoV-2-specific activated cells at 10 months were mainly of the effector memory (CD45RO^+^/CCR7^-^) (TEM) subset. Interestingly, however, a 4.7-fold increase of centralmemory T cell frequencies (CD45RO^+^/CCR7^+^) (T_CM_) was found 10 months after symptom onset (from 3.7% at T3 to 17.6% at T4; P=0.002) (**Figure 6D**).

Only in adults, the frequency of S-SARS-CoV-2-specific IFNγ-producing T cells was still correlated with S1-SARS-CoV-2-specific serum IgG concentrations at 10 months after symptom onset [r_s_=0.64 (P=0.004)] (**Figure 6E**). S1-SARS-CoV-2-specific IgG serum antibody concentrations significantly decreased at 10 months compared to earlier time points in both children and adults [median IgG concentrations of 10.8 AU/mL at T4 versus 24.6 AU/mL at T2 (P=0.0053) in children, and 18.7 AU/mL at T4 versus 35.8 AU/mL T3 versus in adults (P=0.0002)]. Despite this decline, 90% (18/20) of infected children and 89% (16/18) of infected adults remained S1-SARS-CoV-2-IgG seropositive 10 months after infection. No differences were observed in anti-S1-SARS-CoV-2 serum IgG concentrations at 10 months between children and adults (**Figure 6F**).

## Discussion

Most infections with SARS-CoV-2, especially among children, have a mild course. But, do children, despite experiencing mild infection, develop memory T cell immunity? Here, we describe the kinetics, function, and phenotype of SARS-CoV-2-specific T cells of infected children in comparison with adults experiencing mild to moderate COVID-19 symptoms. Only limited studies have been reported investigating the immune responses in children with mild/asymptomatic SARS-CoV-2 infection (17‐21). The strength of our study is that we evaluated the recall T cell response upon SARS-CoV-2 specific stimulation, and compared it to unexposed children and adults.

First, we analyzed immune cell populations over time after infection. Frequencies of total T cells, B cells, monocytes, or NK cellswere comparable between healthy age-matched control groups and infected groups. However, in infected adults, slightly higher frequencies of CD4^+^ T_EM_ and CD8^+^ T_EM_ cell subsets were found at later time points after infection. This small increase in TEM cell subsets may be caused by an increase of circulating SARS-CoV-2-specificCD4^+^ T_EM_ and CD8^+^ T_EM_ cells in response to SARS-CoV-2 infection. In line with this, in infected adults also transiently higher frequencies of CD4^+^ T_CM_ and T_EMRA_ cells expressing CD38/HLA-DR were observed early after infection,and inbothinfectedchildren and infected adults, transiently higher frequencies of CD8^+^ T cells expressing CD38/HLA-DR were observed within the T_CM_/T_EM_/T_N_ cell subsets at the earlier time points after infection. This indicatesthat antigen-specific activation of T cells was triggered in adults as well as in children by SARS-CoV-2 infection. T cell activation, especially robust CD38^+^/HLA-DR^+^CD8^+^T cell responses, has been identified as a hallmark of acute COVID-19 (2, 4, 8).

We found higher frequencies of SARS-CoV-2-specific IFN-Γ^+^ T cells in all infected groups compared to the unexposed control groups upon any of the three antigenic stimulations that included heat-inactivated SARS-CoV-2, and overlapping peptides of SARS-CoV-2 spike protein (S-SARS-CoV-2), and nucleocapsid protein (N-SARS-CoV-2). Ingeneral, frequencies of IFN-γ^+^-T cells reactive against SARS-CoV-2 antigens were lower in infected children, who generally had mild/asymptomatic infection, compared to infected adults. The lower SARS-CoV-2-specific T cell response observed in children suggests that other compartments of the immune system, such as the innate immune response, contribute to faster clearing of the infection, as demonstrated by others (31–33). The higher T cell responses in infected adults could largely be explained by higher T cell responses found in severalmoderate cases, although adultswith mild complaints also tended to have slightly higher responses than infected children. This is in agreement with findings from other studies showing higher frequencies of SARS-CoV-2-specific T cells in severe patients compared to mildly symptomatic patients (8, 15). In contrast, critically ill patients have been reported to exhibit qualitatively impaired S-SARS-CoV-2-specific CD4^+^ T cell responses, indicating that a good CD4_+_ T cell response may protect against serious disease (15). In agreement with our IFN-γ ELISPOT data, significantly lower frequencies of SARS-CoV-2-specific activated CD25^+^ CD137^+^ CD4^+^ T cells were observed in infected children compared to infected adults. Recently, Cohenet al. also described that acute and memory CD4^+^ T cells in SARS-CoV-2-infected children were significantly lower than in adults, while polyfunctional cytokine production byT cells was comparable (21). Furthermore, in accordance with Cohen et al. (21), we found that the SARS-CoV-2 activated T cells mainly belonged to the CD4^+^ effector memory subset (T_EM_: CD45RO^+^/CCR7^-^) in our experimental setting. Data on the SARS-CoV-2-specific IFN-γ^+^ T cell response and CD4^+^ T cell activation correlated, suggesting that IFN-γ was produced by antigen-specific CD4^+^ T cells. Apart from IFN-γ, SARS-CoV-2-specific T cells produced IL-2, suggestive for a Th1 phenotype of the CD4^+^ T_EM_.

It has been demonstrated that SARS-CoV-2-specific T cell responses can be retained 6-8 months following infection regardless of disease severity (8, 16, 34). Although, other studies showed a clear decline of the T cell response over a 6-8 month period in asymptomatic as well as symptomatic COVID-19 patients (35, 36). In our study, at 10 months after symptom onset, the frequencies of SARS-CoV-2-specific IFN-γ^+^ T cells were significantly reduced in adults and were nearly undetectable in children. Whether this is a result of waning immunity or migration of memory SARS-CoV-2-specific T cells to peripheral tissues remains to be investigated. Interestingly, the memory phenotype of the activated SARS-CoV-2-specific CD4^+^ T cells in adults showed a shift from mainly T_EM_ to a more T_CM_ phenotype at 10 months after infection; T_CM_ subset increased from 3.7% to 17.6% of the antigen-specific CD4^+^ T cells. This T_CM_ subset may persist for longer after infection.

In contrast to the observed SARS-CoV-2-specific CD4^+^ T cell response, we found very low frequencies of activated (CD25^+^/CD137^+^) CD8^+^ T cells upon SARS-CoV-2-specific stimulation. An explanation for this may be that CD8^+^ T cells have migrated to the local sites of infection to attack virus-infected cells or might be a result of the antigenic stimulation used in our study. Smaller peptides (9 to 10-mers instead of 15-mers) or live SARS-CoV-2 may be more suitable to measure CD8^+^ T cell responses. Sekine et al. also observed proportionately larger SARS-CoV-2-specific CD4^+^ T cell responses than CD8^+^ T cell responses to different sets of overlapping peptides in the convalescent phase of both mild and severe COVID-19 cases, although in that study also IFN-γ^+^ CD8^+^ T cell responses were detected (8).

We found positive correlations between SARS-CoV-2-specific IFN-γ^+^ T cell frequency and serum-IgG, -IgM, and -IgA antibody concentrations to S1-SARS-CoV-2 in both children and adults. Although, it should be taken into account that the antibody concentrations of considerable numbers of children were below the threshold for seropositivity (10). Other studies have also shown positive correlations between S-SARS-CoV-2-specific IgG antibodies and T cell responses (6, 37, 38).

Pre-existing cross-reactive T cell immunity generated by common cold human coronaviruses (HCoV) has been suggested to affect clinical outcomes of SARS-CoV-2 infection. T cell lines from unexposed healthy donors specific for S-HCoV-229E and -OC43 were cross-reactive to S-SARS-CoV-2 (39). Based on these findings, the authors suggested that children may have higher HCoV prevalence due to more frequent social contacts, explaining their lower risk for severe COVID-19 (39). In the present study, we did, however, not find a significant difference in the low frequencies of S-HCoV-OC43-reactive IFNγ^+^ T cells between unexposed children and unexposed adults. In another study with mild COVID-19 adult patients, also low T cell frequencies recognizing S-HCoV-229E/S-HCoV-OC43 peptide pools were found (15). It cannot be excluded that preexisting cross-reactive immunity to other conserved parts of SARS-CoV-2 played a role, or that pre-existing immunity to other HCoV played a role. We, however, found no evidence that pre-existing S-HCoV-OC43-reactive T cells boosted upon SARS-CoV-2 infection could explain the mild course of infection.

Limited data is available on the immune response to SARS-CoV-2 in children. Here, we show that infected children do develop a SARS-CoV-2-specific T cell response. However, frequencies of SARS-CoV-2 reactive T cells were lower in infected children who almost all had mild/asymptomatic infection compared to infected adults with mild to moderate COVID-19. Predominantly CD4^+^ T cells, and not CD8^+^ T cells, were activated upon stimulation with SARS-CoV-2 antigens. Nevertheless, frequencies of SARS-CoV-2-specific T cells circulating in the blood were significantly reduced at 10 months after infection, although migration of memory SARS-CoV-2-specific T cells to peripheral tissues may have taken place. Importantly, our data indicate that an antigen-specific T cell and antibody response is developed after experiencing mild SARS-CoV-2 infection. It remains to be elucidated to what extent this SARS-CoV-2-specific response can contribute to an effective recall response after reinfection.

## Data Availability Statement

Datasets are available on request: The raw data supporting the conclusions of this article will be made available by the authors upon request, with consideration of the participants’ privacy rights.

## Ethics Statement

The studies involving human participants were reviewed and approved by Medical-Ethical Review Committee (MERC) of University Medical Center Utrecht (Netherlands Trial Register (https://www.trialregister.nl/): NL9850, MERC Reference number: NL13529.041.06). Protocols for the cohort studies with unexposed children (Immfact, NTR: NL9775, MERC Reference number: NL4679.094.13) and adults (NVI-255, NTR: NL1952, MERC Reference number: NL29241.000.09) were approved by Medical-Ethical Review Committees of the Netherlands. Written informed consent to participate in this study was provided by all participants and/or from parents/guardians of minor participants (<16 years old).

## Author Contributions

DR, NR, JB, and LB designed and conducted the clinical study. AM conducted and analyzed data of the PCR testing. JW, PK, ME, MP, AC, and CE designed, conducted, and acquired data of T cell immunity testing. GH and RS designed, conducted, and acquired data of antibody testing. VP and PK statistically analyzed data. VP and ME made figures. PK wrote the first draft of the manuscript and all authors contributed to the final version of the paper. All authors contributed to the article and approved the submitted version.

## Funding

This work was supported by the Dutch Ministry of Health, Welfare, and Sport.

## Conflict of Interest

The authors declare that the research was conducted in the absence of any commercial or financial relationships that could be construed as a potential conflict of interest.

## Publisher’s Note

All claims expressed in this article are solely those of the authors and do not necessarily represent those of their affiliated organizations, or those of the publisher, the editors and the reviewers. Any product that may be evaluated in this article, or claim that may be made by its manufacturer, is not guaranteed or endorsed by the publisher.
