# Supplementary material for: Children and Adults With Mild COVID-19: Dynamics of the Memory T Cell Response up to 10 Months
Source: Front Immunol. 2022 Feb 7;13:817876. doi: 10.3389/fimmu.2022.817876 (PMC8858984; doi:10.3389/fimmu.2022.817876)
Supplement: Supplementary file 1 [file DataSheet_1.docx]

Supplementary Material

Children and adults with mild COVID-19: dynamics of the memory T cell response up to 10 months

*Patricia Kaaijk, Verόnica Olivo Pimentel, Maarten Emmelot, Martien Poelen, Alper Cevirgel, Rutger Schepp, Gerco den Hartog, Daphne Reukers, Lisa Beckers, Josine van Beek, Cécile van Els, Adam Meijer, Nynke Rots, Jelle de Wit*


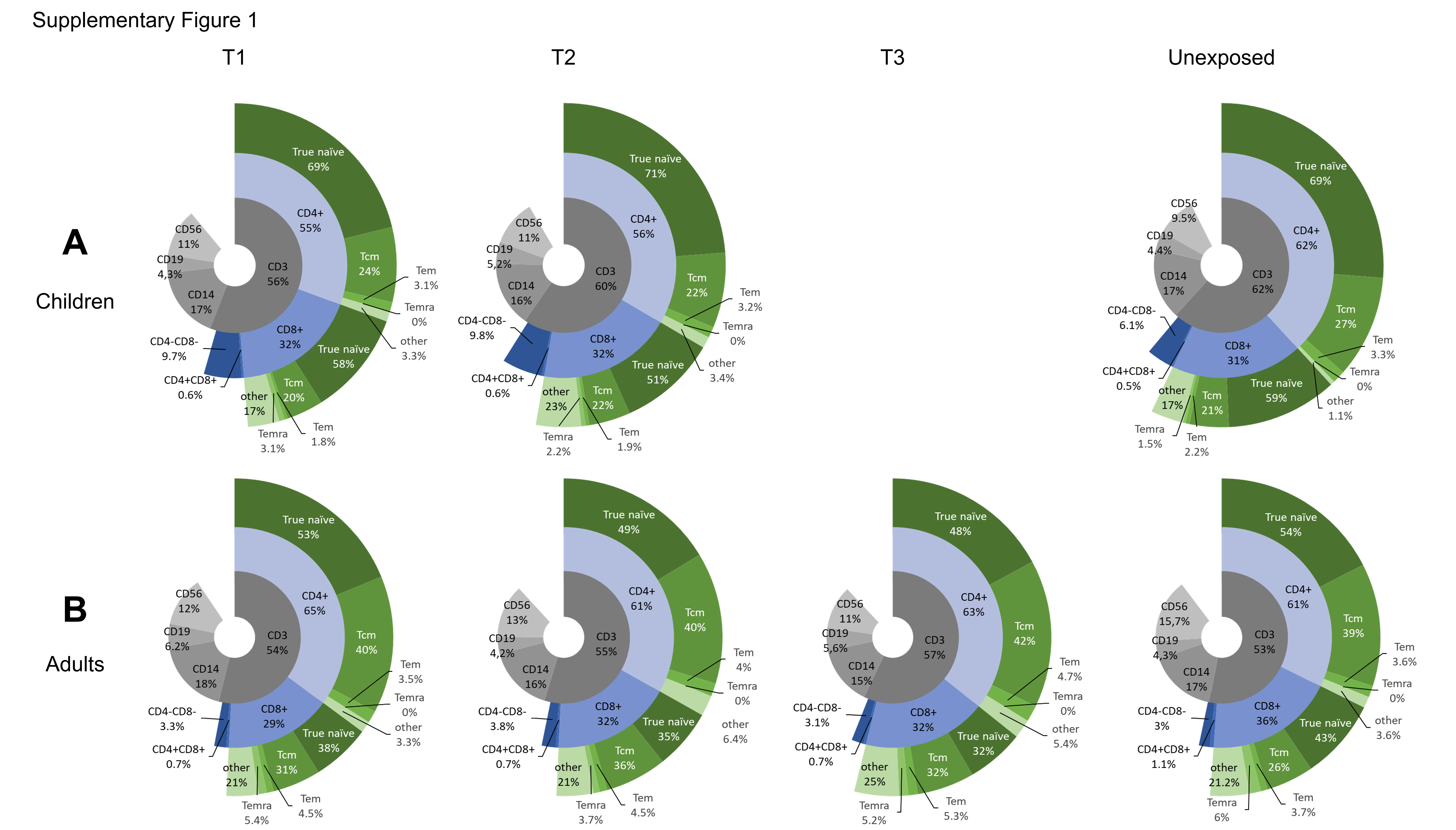


**Supplementary Figure 1. *Ex vivo* immune profiling of PBMCs of adults and children over time after infection**

Sunburst plots showing the results of immunophenotyping of PBMCs performed with multicolor flow cytometry by staining for: (inner circle, lymphocyte gate) B cells (CD19^+^, CD3^-^); NK cells (CD56^+^, CD3^-^, CD19^-^); CD14^+^ monocytes (CD14^+^, CD3^-^, CD19^-^, CD56^-^), T cells (CD3^+^); (middle circle, CD3^+^ T cells) CD4^+^ T cells (CD4^+^, CD8^-^); CD8^+^ T cells (CD8^+^, CD4^-^); CD4^+^/CD8^+^ T cells (CD3^+^, CD4^+^); CD4^-^/CD8^-^ T cells (CD4^-^, CD8^-^); (outer circle) CD4^+^ and CD8^+^ memory T cell subsets are identified based on CD45RO, CCR7, CD27, CD28 and CD95 staining (true naive, CD45RO^-^, CD27^+^, CCR7^+^, CD95^-^; TCM, CD45RO^+^, CD27^+^; T_EM_, CD45RO^+^, CD27^-^; T_EMRA_, CD45RO^-^, CD27^-^, CD28^-^, CD57^+^), as indicated. Children in time after SARS-CoV-2 infection and unexposed healthy children (A); Adults in time after SARS-CoV-2 infection and unexposed healthy adults (B). Data are presented as median percentages of presented cell types. Presented median percentages of T cell subsets in the sunburst plots may slightly differ from the median percentages described in the text as for comparison between the various time points, as described in the text, only paired data were considered. T1, first timepoint of sampling for adults median 12.5 days and children median 8 days post-symptom onset; T2, 10-14 days after T1; T3, 4-6 weeks after T1.

**Supplementary Figure 2. *Ex vivo* CD38 and HLA-DR expression on CD4^+^ and CD8^+^ T cells over time after SARS-CoV-2 infection**

Flow cytometry using Trucount tubes was performed on fresh blood to analyze CD4^+^ and CD8^+^ T cells expressing CD38 and/or HLA-DR as markers for antigen-specific T cells. Dot plots are presented showing the expression of CD38/HLA-DR in CD4^+^ T cell subsets (upper panel) and CD8^+^ T cell subsets (lower panel) in (A) children and B (adults) in time after infection.

Each dot represents one subject. Bars indicate median percentage of CD38^+^/HLA-DR^+^ cells within the T cell subsets. Differences were compared using the Wilcoxon signed-rank test (for comparison of two paired groups) (infected children at T1 versus T2) or the Friedman test with Dunn’s multiple comparison tests (infected adults at T1 versus T2 versus T3). Statistically significant comparisons are indicated, with P values < 0.05 considered significant. T1, first timepoint of sampling for adults median 12.5 days and for children median 8 days post-symptom onset; T2, 10-14 days after T1; T3, 4-6 weeks after T1.


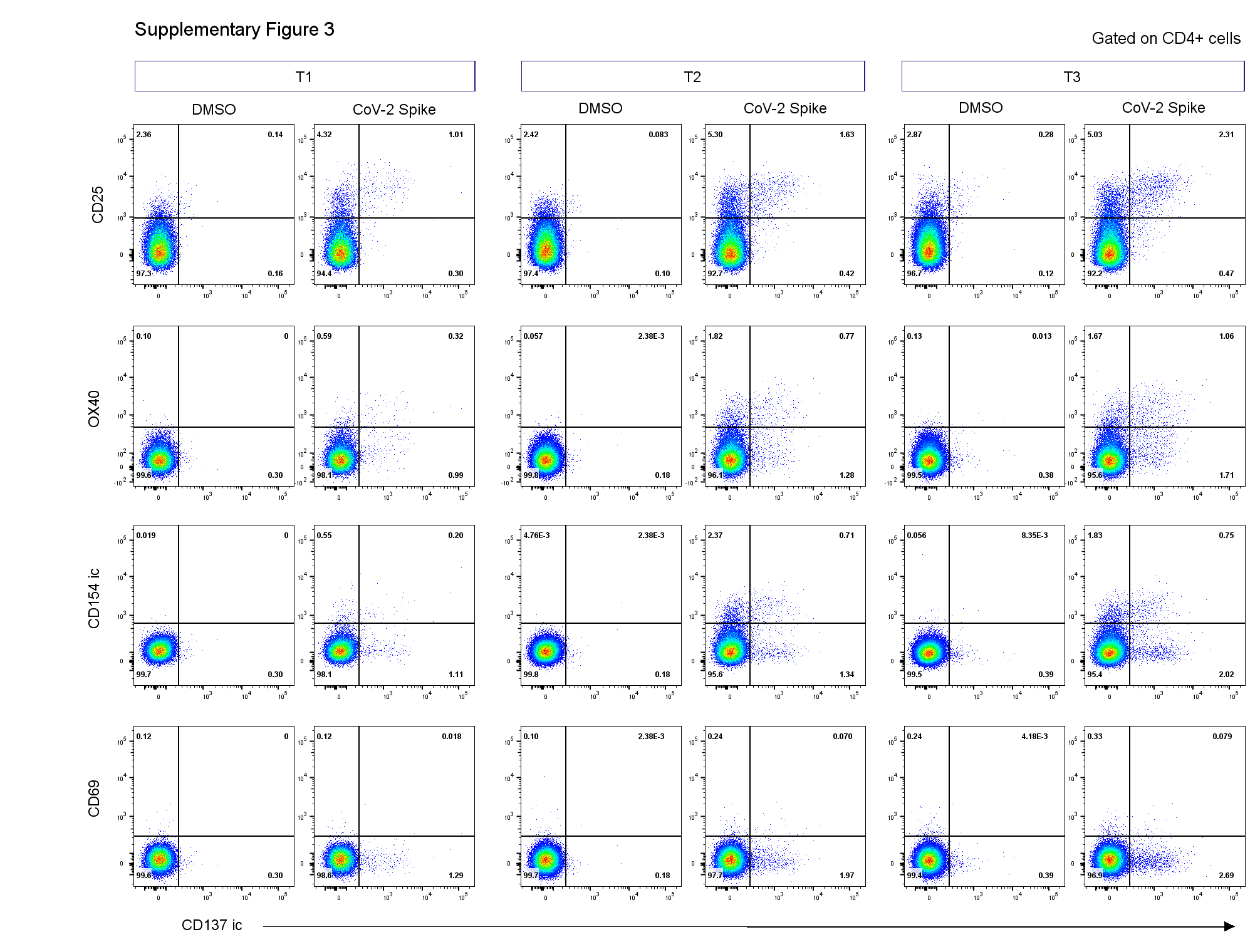


**Supplementary Figure 3. Expression of different activation markers on spike-SARS-CoV-2-stimulated CD4^+^ T cells**

Representative flow cytometry plots of expression of various activation markers on gated CD4^+^ T cells 24 hours after incubation of PBMCs from infected adults with overlapping peptides of SARS-CoV-2 spike protein versus DMSO (negative control) in time after infection, T1 (left panel), T2 (middle panel), and T3 (right panel). The following activation markers were used: CD25, OX40, CD154 (intracellular staining), CD69, and CD137 (intracellular staining). T1, first timepoint of sampling for adults median 12.5 days and children median 8 days post-symptom onset; T2, 10-14 days after T1; T3, 4-6 weeks after T1

**Supplementary Figure 4. Percentages of activated CD8^+^ T cells in adults and children over time after infection versus unexposed healthy controls**

Dot plots summarizing the frequencies of CD25^+^/CD137^+^ activated CD8^+^ T cells responding to SARS-CoV-2 and HCoV-OC43 antigens for children versus unexposed controls (left panel), adults versus unexposed controls (right panel) in time after infection. Frequencies of CD25^+^/CD137^+^ activated CD8^+^ T cells responding to (A) inactivated SARS-CoV-2, (B) overlapping peptides of SARS-CoV-2 spike protein, (C) overlapping peptides of SARS-CoV-2 nucleocapsid protein, and (D) overlapping peptides of HCoV-OC43 spike protein. Each dot represents one subject. Bars indicate median of % CD25^+^/CD137^+^ CD8^+^ T cells. For unpaired comparisons, Kruskal-Wallis rank-sum test with Dunn’s posthoc test for multiple comparisons was used (infected children at T1 or T2 versus unexposed children, infected adults at T1 or T2 versus unexposed adults). Differences between paired data were compared using the Wilcoxon signed-rank test (for comparison of two paired groups) (infected children at T1 versus T2) or the Friedman test with Dunn’s multiple comparison tests (infected adults at T1 versus T2 versus T3). Statistically significant comparisons are indicated, with P values < 0.05 considered significant. T1, first timepoint of sampling for adults median 12.5 days and children median 8 days post-symptom onset; T2, 10-14 days after T1; T3, 4-6 weeks after T1.
